# Seeing pre-screened, regular clients associated with lower odds of workplace sexual violence and condom refusal amidst sex work criminalization: findings of a community-based cohort of sex workers in Metro Vancouver, Canada (2010-2019)

**DOI:** 10.1186/s12889-022-12903-9

**Published:** 2022-03-17

**Authors:** Bronwyn McBride, Kate Shannon, Jennie Pearson, Andrea Krüsi, Melissa Braschel, Shira M. Goldenberg

**Affiliations:** 1 Centre for Gender & Sexual Health Equity, c/o St. Paul’s Hospital, 1081 Burrard St, Vancouver, BC V6Z1Y6 Canada; 2 Faculty of Medicine, University of British Columbia, 317-2194 Health Sciences Mall, Vancouver, BC V6T1Z3 Canada; 3 Faculty of Health Sciences, Simon Fraser University, 8888 University Drive, Burnaby, BC V5A1S6 Canada; 4 Division of Epidemiology and Biostatistics, School of Public Health, San Diego State University, Hardy Tower – Room 119, 5500 Campanile Drive, San Diego, CA 92182-4162 USA

**Keywords:** Sex work, Sex work clients, Condom use, Sexual violence, End-demand

## Abstract

**Background:**

Research that accurately represents how characteristics of sex work clients relate to sex workers’ labour conditions is crucial for informing evidence-based legislation which upholds sex workers’ human rights. As little quantitative research has examined how seeing regulars (repeat clients) impacts sex workers’ occupational safety, particularly under ‘end-demand’ criminalization in Canada, our study aimed to explore how seeing mostly regulars shapes workplace sexual violence and client condom refusal.

**Methods:**

We drew on longitudinal data from a community-based open cohort of 900+ sex workers in Vancouver, recruited via time-location sampling during day and late-night outreach to indoor, outdoor, and online solicitation spaces. We used logistic regression analyses and multivariable GEE confounder models to 1) describe correlates of seeing mostly pre-screened, regular clients, 2) identify associations between seeing mostly regulars and odds of experiencing occupational outcomes of workplace sexual violence and client condom refusal, and 3) examine the interaction between seeing mostly regulars and work environment on workplace sexual violence and client condom refusal.

**Results:**

Participants’ median age was 35, and 55.6% had completed high school. Over the 9-year study (*n*=925), 20.9% (193) experienced 282 events of workplace sexual violence and 40.2% (372) faced 702 events of client condom refusal. In multivariable GEE confounder models, seeing mostly regulars was associated with reduced odds of sexual violence (AOR 0.73, 95%CI 0.53-1.02, *p*=0.067) and client condom refusal (AOR 0.70, 95%CI 0.57-0.86). In multivariable GEE confounder models examining the additive interaction between seeing mostly regulars and work environment, participants who saw mostly regulars and primarily worked in outdoor or informal indoor venues faced significantly lower odds of experiencing workplace sexual violence (AOR 0.69, 95%CI 0.49-0.95) and client condom refusal (AOR 0.64, 95%CI 0.52 -0.80) relative to those who worked in the same venues and did not see mostly regulars.

**Conclusion:**

Our findings highlight protective effects of seeing pre-screened regulars within a criminalized setting. Removal of ‘end-demand’ client criminalization is needed to enable sex workers to effectively screen clients, support HIV/STI prevention, and advance sex workers’ human rights.

## Background

In criminalized settings globally, sex workers face labour rights violations including high levels of workplace violence perpetrated by aggressors and police: a 2014 global systematic review identified a lifetime prevalence of physical, sexual or combined workplace violence against women sex workers from 45% to 75% [1]. While individuals who purchase sex services are commonly represented in policy discourse as violent, exploitative, and contributors to HIV (human immunodeficiency virus)/STI (sexually transmitted infection) burden, a broadening body of evidence has highlighted the criminalization of aspects of sex work as a major determinant structuring sex worker and client interactions, with impacts on condom use negotiation and sex workers’ exposure to workplace sexual violence (defined as sexual assault or rape in the context of work) [1–5]. Amid prominent stereotypes of sex work clients as coercive and/or violent and a wave of ‘end-demand’ approaches to sex work legislation [6], purchasing sex services is currently criminalized in 75 countries globally [7]. Research that accurately represents characteristics of sex work clients and how these relate to sex workers’ labour conditions is crucial for informing evidence-based sex work legislation, which is a well-established determinant of sex workers’ occupational health and human rights [8–10]. However, particularly in North America and many European countries where end-demand laws have been implemented, evidence on the implications of seeing mostly regular clients (pre-screened, repeat clients) on sex workers’ occupational safety remains limited.

Existing literature shows that client-sex worker interactions are shaped by structural determinants (i.e., laws, policies), work environment factors (i.e., outdoors vs. various indoor spaces, working alone vs. with colleagues or a manager), and interpersonal factors (i.e., gendered power dynamics, patriarchal ideologies, racism) [11–16]. Qualitative studies have documented diverse client-sex worker dynamics in terms of occupational health issues such as workplace violence and condom use: a Canadian qualitative study involving indoor sex workers found that 68% of participants (74 of 109) reported experiencing no violence in their work [17], while a study among street-based sex workers in Baltimore found that 20% (7 of 35) reported facing sexual violence from clients in the past month, most commonly in the context of condom use negotiation [18]. A US study involving sex work clients found that most clients sought paid interactions which mirrored non-remunerative relationships, and reported penile-vaginal sex with a condom, oral sex without a condom, and deep kissing as the most common activities engaged in with a sex worker [19]. In research conducted in the Caribbean, Asia, sub-Saharan Africa, greater relationship intimacy between sex workers and regular clients has been linked to lower condom use: a quantitative study in the Dominican Republic involving sex workers and clients found that higher relationship intimacy (evaluated using a 9-item measure including perceived levels of trust, affection, and love) was negatively associated with condom use in multivariable analysis (AOR 0.25, 95% CI 0.14-0.45) [20], while qualitative studies in Jamaica, Madagascar, Nepal and Indonesia found that having an established relationship, repeat dates, and viewing the client as more of a boyfriend were linked to lower and inconsistent condom use [21–24]. However, limited epidemiological research has explored the implications of seeing regular clients on condom use negotiation among sex workers in global northern contexts such as Canada. This gap is particularly salient under laws implemented in 2014 which criminalized purchasing sex services for the first time in Canadian history.

In the 1990s, North American criminal justice institutions began to shift focus from targeting sex workers towards targeting clients with the aim of discouraging men from seeking sex services [25]. The “end-demand” legislative model (Nordic/abolitionist model) was first implemented in Sweden in 1999 [26], and subsequently in Norway, Iceland, France and several other countries [6]. Based on ideologies depicting sex workers as victims and clients as abusers who exploit vulnerable women (including through coercing condomless sex), end-demand laws typically criminalize the purchase of sex [26], while leaving the sale of sex legal to lessen the criminalization of sex workers. However, criminalizing any aspect of sexual service exchange can impact occupational health including sexual health and HIV/STI exposure in sex worker-client interactions, as punitive policing has been robustly documented to push sex workers and clients into isolated settings, increase workers’ vulnerability to violent perpetrators, and limit their negotiating power for condom use [5, 10, 14, 27–29]. Concerningly, limited research from Sweden and France suggests that end-demand laws undermine sex workers’ ability to screen new clients (the process of collecting client information; vetting clients; and establishing boundaries, services offered, prices, and use of personal protective equipment), as clients fear providing identifying information due to the potential consequences of criminalization [28, 30–32]. End-demand laws have also been shown to undermine condom use negotiation, enhance exposure to workplace violence, and heighten barriers to voluntary HIV/STI testing [28, 30, 31].

In Canada, end-demand legislation was enacted in December 2014 [33] after the Supreme Court declared the previous sex work laws unconstitutional for violating sex workers’ rights to security of person [34]. This legislation explicitly criminalizes the purchase of sex services in all circumstances, and also criminalizes third parties (i.e., venue owners/managers, security) who gain material benefits from others’ sex work [35]. Concerningly, emerging research suggests that Canadian end-demand laws reproduce the unsafe labour conditions of the previous unconstitutional legislation by reducing sex workers’ ability to screen clients, limiting access to condoms in sex work venues, and restricting sex workers’ ability to call police for protection in the event of facing workplace sexual violence or theft [36, 37]. A qualitative study in Vancouver found that police targeting clients of street-based sex workers undermined sex workers’ existing client screening and safety strategies, heightened their exposure to workplace violence, and disrupted relationships with their pre-screened regular clients [14]. Amid end-demand laws which criminalize the purchase of sex for the first time in Canadian history, epidemiological research on sex workers’ interactions with regular clients and how this shapes occupational health and safety outcomes is urgently needed.

### Study objectives

Adapting a structural determinants framework [8], we used prospective cohort data collected over a 9-year study period to 1) describe correlates of seeing mostly pre-screened, regular clients, 2) identify independent associations between seeing mostly regulars and odds of experiencing occupational outcomes of workplace sexual violence and client condom refusal, and 3) examine the interaction between seeing mostly regulars and work environment on workplace sexual violence and client condom refusal, over 9 years.

## Methods

Longitudinal data were drawn from a community-based open prospective cohort, An Evaluation of Sex Workers Health Access (AESHA) which initiated recruitment in 2010 and is based on community collaborations since 2005. Eligibility criteria at baseline include identifying as a cisgender or transgender woman, having exchanged sex for money in the last month, aged 14+, and able to provide written informed consent. Time-location sampling supported recruitment through daytime and late-night outreach to diverse outdoor (i.e., streets, alleys), indoor settings (i.e., massage parlours, micro-brothels, hotels, bars) and online solicitation spaces across Metro Vancouver. Following an open cohort design, sex workers continue to be actively recruited. Annual retention of participants under active follow-up is >90%, and primary reasons for attrition include mortality and migration outside Metro Vancouver. Extensive efforts are made to continue to follow sex workers who move outside Metro Vancouver during the study, including mobile outreach/interview teams and phone interviews, to support high retention rates. Since inception, current/former sex workers are hired throughout the project, from interviewers/outreach workers and sexual health research nurses to coordinators. Further detail on AESHA’s community origins is available elsewhere [38].

After obtaining informed consent, participants completed questionnaires administered by a trained interviewer in English, Cantonese or Mandarin at baseline and semiannual follow-up visits. The AESHA questionnaire elicited responses on socio-demographics, work environments, structural factors, and health access and outcomes, and draws extensively on published scales as well as community-informed measures. Interviews were conducted at study offices in Vancouver or a confidential space of participants’ choice (e.g., home, work). Data are securely collected and managed using REDCap [39] electronic data capture tools hosted at the University of British Columbia. Participants receive voluntary HIV/STI/HCV (hepatitis C virus) serology testing by a project nurse and are offered treatment onsite, if needed, for symptomatic STIs and Papanicolaou testing, regardless of enrolment in the study. All participants received $40 CAD at each biannual visit. The study holds ethical approval through Providence Health Care/University of British Columbia and Simon Fraser University Research Ethics Boards.

### Measures

#### Primary variable and outcomes

This study used a time-updated, binary measure of seeing mostly pre-screened, regular clients, defined as participants reporting that >75% of their clients in the last six months were repeat clients. Those for whom ≤75% of their clients were repeat clients were coded as not seeing mostly regulars. This was used as the outcome variable in Objective 1, and as the primary exposure of interest in Objectives 2 and 3. Time-updated occupational health and safety outcomes of interest included 1) workplace sexual violence (defined as sexual assault or rape in the context of work, perpetrated by aggressors posing as clients) and 2) client condom refusal (defined as being coerced into oral/vaginal/anal sex without a condom by any client) in the last six months.

#### Independent variables

Adapting a structural determinants framework [8], independent variables at individual and structural levels were explored. Time-fixed variables included race (white, Indigenous, or woman of colour [i.e., Black, Asian, Latina]) and high school completion (vs. less than high school). All other variables were time-updated at each semiannual follow-up, examining events during the past six months or current measures at the study visit. *Individual factors* included age, non-injection drug use (e.g., cocaine, crystal meth; excluding cannabis and alcohol use), and injection drug use.

*Structural determinants* included migration (migrated within the past 5 years, migrated over 5 years ago, or Canadian-born), *housing* (unstable housing, defined as any stays in single-room occupancy hotels/supportive housing or staying with family/friends; homelessness), and *income* (average weekly income from sex work; financially supporting dependents). *Work environment* included primary place serving clients (informal indoor space [e.g., apartment, hotel, client’s place], formal indoor venue [e.g., managed, in-call establishment such as a massage parlour], or outdoor/public space [e.g., street, car]); and average number of clients per month.

*Policing* measures included being arrested while working; and facing police harassment while working (police raid, police parked nearby/drove by repeatedly, told to move on, threatened with arrest/detainment/fines, being searched/followed/picked up and driven elsewhere to work, verbally harassed, detained, physically assaulted, drugs/drug use equipment confiscated, searched for condoms/condoms taken, other property taken, or propositioned to exchange sex/coerced into providing sexual favours). *Health* measures included facing any barriers to health care (e.g., long wait times, health insurance barriers, poor treatment by health care professionals) and having had an HIV test (vs. no test vs. living with HIV). Lastly, we evaluated exposure to the time period surrounding implementation of Canadian end-demand legislation (2010–2013 vs. 2015–2017). As the end-demand bill was introduced in January 2014 and passed into law in December 2014, this time period was excluded from all analyses due to potential variation in law enforcement. The first three months of 2015 were also excluded to account for outcome measures referring to the preceding six months. Additionally, as not all participants do sex work at every follow-up visit, analyses were restricted to observations where participants did sex work in the last six months.

### Objective 1: Correlates of seeing mostly pre-screened regular clients

For objective 1, we examined baseline descriptive statistics for explanatory variables stratified by the outcome of seeing mostly pre-screened, regular clients. Frequencies and proportions were calculated for categorical variables and measures of central tendency and dispersion (i.e., mean, median, interquartile range (IQR)) for continuous variables. Differences were assessed using the Wilcoxon rank-sum test for continuous variables and Pearson’s chi-square test (or Fisher’s exact test for small cell counts) for categorical variables. We then conducted bivariate and multivariable logistic regression using generalized estimating equations (GEE) and an exchangeable correlation matrix to account for repeated measures on the same participants. Variables significantly associated with seeing mostly regulars at *p*<0.10 and those hypothesized to be associated a priori were considered for inclusion in the multivariable explanatory model. The best fitting multivariable model, as indicated by the lowest quasi-likelihood under the independence model criterion, was determined using a backward stepwise selection process. All analyses were performed in SAS version 9.4 (SAS, Cary, NC) and all *p*-values are two-sided.

### Objective 2: Independent associations between seeing mostly regulars and odds of experiencing workplace sexual violence and client condom refusal

For objective 2, we constructed two separate multivariable GEE confounder models to assess the independent associations between seeing mostly pre-screened regulars and odds of experiencing 1) workplace sexual violence and 2) client condom refusal, in the last 6 months. For the confounder models, our aim was to find the most parsimonious model while adjusting for confounders that could bias results. Variables from the best fitting multivariable explanatory model for seeing mostly regulars were considered potential confounders. The most parsimonious models were determined using the process described by Maldonado and Greenland [40], in which potential confounders were removed in a stepwise manner, and variables that altered the association of interest by <5% were systematically removed from the model.

### Objective 3: Interaction between seeing mostly regulars and work environment on workplace sexual violence and client condom refusal

For objective 3, we developed an additive interaction variable between seeing mostly pre-screened regular clients and work environment (primary place serving clients), which was time-updated to capture events in the last six months at each visit. For this sub-analysis, work environment was examined as a binary variable (formal indoor venues vs. outdoor or informal indoor space) for ease of interpretation and statistical power. The additive interaction variable included four categories: saw mostly regular clients in a formal indoor venue, did not see mostly regulars in a formal indoor venue, saw mostly regular clients in an outdoor/public or informal indoor space, and did not see mostly regular clients in an outdoor/public or informal indoor space (the reference category). We constructed two separate multivariable GEE confounder models to examine the additive interaction between seeing mostly regulars and work environment on occupational outcomes of 1) workplace sexual violence and 2) client condom refusal. Potential confounders identified in Objective 2 were included in the full multivariable models. As above, variables that altered all of the associations of interest by <5% were systematically removed from the model. The statistical significance was set at *p* value <0.1 for inclusion of variables in final models. Power calculations conducted for the full cohort suggested that maintaining a sample size of *N*=800 is necessary to ensure sufficient statistical power for our primary endpoints of HIV/STI incidence and risks, which is anticipated to provide sufficient power for less rare outcomes related to occupational conditions and client dynamics.

## Results

### Objective 1: Correlates of seeing mostly pre-screened regular clients

Analyses included 4045 observations amongst 925 sex workers between Jan 2010 – Feb 2019. Participants completed a median of 3 visits (IQR 1-6). The drop-out rate for the cohort is approximately 13% per year, with death accounting for 8% of dropouts. We use an open cohort design to maintain sample size over time, and recruit about 9% per year. Over the 9-year study, 20.9% (193) experienced 282 events of workplace sexual violence and 40.2% (372) faced 702 events of client condom refusal. At baseline, 37.8% of participants primarily served clients in outdoor/public spaces, 29.3% in informal indoor spaces (e.g., apartment, hotel, client’s place), and 30.8% in formal indoor venues (e.g., massage parlours). 31.2% (289) had experienced recent police harassment while working. Baseline descriptive statistics are presented in Table 1.

**Table 1:** Baseline individual and structural factors stratified by seeing mostly regular clients among sex workers in Metro Vancouver (n=925), AESHA 2010-2019

Characteristic	Total (***N*** = 925) n (%)	Saw mostly regular clients^a^		***P***
		Yes (*N*=228) n (%)	No (*N*=697) n (%)	
**Individual factors**				
Age, median (IQR)	35 (28-42)	36 (29.5-43)	35 (28-42)	0.059
Completed high school	514 (55.6)	132 (57.9)	382 (54.8)	0.415
Non-injection drug use^a^	615 (66.5)	168 (73.7)	447 (64.1)	0.007
Injection drug use^a^	374 (40.4)	102 (44.7)	272 (39.0)	0.127
**Structural determinants**				
Time since im/migration^b^				
Non-im/migrant	659 (71.2)	183 (80.3)	476 (68.3)	
Im/migrated ≤5 years ago	99 (10.7)	6 (2.6)	93 (13.3)	
Im/migrated >5 years ago	147 (15.9)	35 (15.4)	112 (16.1)	<0.0001
Race				
White	288 (31.1)	78 (34.2)	210 (30.1)	
Indigenous	354 (38.3)	107 (46.9)	247 (35.4)	
Woman of colour	283 (30.6)	43 (18.9)	240 (34.4)	<0.0001
*Housing & income*				
Any unstable housing^a^	728 (78.7)	194 (85.1)	534 (76.6)	0.004
Homeless/living on street^a^	279 (30.2)	69 (30.3)	210 (30.1)	0.981
Average weekly income from sex work^a^ ($CAD), median (IQR)	500 (250-1000)	500 (200-1000)	500 (270-1000)	0.028
Currently financially supports dependents	277 (30.0)	78 (34.2)	199 (28.6)	0.105
*Work environment*				
Primary place serving clients^a^				
Outdoor/public space	350 (37.8)	74 (32.5)	276 (39.6)	
Informal indoor space	271 (29.3)	105 (46.1)	166 (23.8)	
Formal indoor venue	285 (30.8)	41 (18.0)	244 (35.0)	<0.0001
Average # of clients/month^a^,^,^ median (IQR)	40 (20-80)	32 (10-60)	48 (24-80)	<0.0001
Client condom refusal^a^	193 (20.9)	44 (19.3)	149 (21.4)	0.562
*Violence & policing*				
Workplace sexual violence^a^	101 (10.9)	23 (10.1)	78 (11.2)	0.644
Police harassment while working^a^	289 (31.2)	60 (26.3)	229 (32.9)	0.063
Arrested/jailed while working^a^	53 (5.7)	12 (5.3)	41 (5.9)	0.724
*Health*				
Experienced any barriers to health care^a^	604 (65.3)	150 (65.8)	454 (65.1)	0.857
HIV testing^a^				
Did not have an HIV test	393 (42.5)	87 (38.2)	306 (43.9)	
Had an HIV test	363 (39.2)	88 (38.6)	275 (39.5)	
Living with HIV	131 (14.2)	46 (20.2)	85 (12.2)	0.011

All data refer to n (%) of participants unless otherwise specified

^a^In the 6 months

^b^immigrant or migrant; inclusive of any immigration status [41]

Over half of participants (57.3%, *n*=530) saw mostly pre-screened regulars at some point, with 1676 events of seeing mostly regulars over the 9-year study. In multivariable GEE analysis, participants who faced homelessness, those who used non-injection drugs, and those working in formal indoor venues (e.g., massage parlours, vs. on the street/in public) had lower odds of seeing regulars (Table 2). Older participants and those working in informal indoor settings (e.g., apartments, vs. on the street/in public) had higher odds of seeing regulars. Participants had a 1.6-fold increased odds of seeing mostly pre-screened regulars (not fewer clients, but a greater proportion of pre-screened clients) after the implementation of end-demand legislation.

**Table 2:** Correlates of seeing mostly regular clients among sex workers in Metro Vancouver (n=925), AESHA 2010-2019

Characteristic	Odds Ratio (95% CI)	Adjusted Odds Ratio (95% CI)
**Individual factors**		
Age (per year older)	1.05 (1.04-1.07)	1.03 (1.02-1.04)
Non-injection drug use^a^	0.91 (0.76-1.08)	0.83 (0.68-1.02)
Completed high school	0.91 (0.74-1.11)	^b^
**Structural determinants**		
Race		
White	ref	
Indigenous	1.08 (0.86-1.37)	
Woman of colour	0.43 (0.33-0.58)	
Time since im/migration		
Non-im/migrant	ref	
Im/migrated ≤5 years ago	0.24 (0.16-0.38)	
Im/migrated >5 years ago	0.49 (0.37-0.65)	
*Housing & income*		
Any unstable housing^a^	1.02 (0.86-1.21)	
Homeless/living on street^a^	0.60 (0.50-0.72)	0.77 (0.65-0.92)
Average weekly income from sex work^a^ (per $100 CAD)	0.94 (0.92-0.95)	
Currently financially supports dependents	1.12 (0.95-1.31)	
*Work environment*		
Primary place serving clients^a^		
Outdoor/public space	Ref	ref
Informal indoor space	3.37 (2.82-4.02)	2.96 (2.48-3.52)
Formal indoor venue	0.73 (0.55-0.96)	0.56 (0.41-0.78)
Average number of clients/month^a^ (per client)	0.99 (0.98-0.99)	
Any inconsistent condom use^a^	0.76 (0.62-0.93)	
*Violence & policing*		
Police harassment while working^a^	0.57 (0.48-0.68)	
Arrested/jailed while working^a^	0.54 (0.33-0.89)	
*Health*		
Experienced any barriers to health care^a^	0.89 (0.78-1.01)	
HIV testing^a^		
Did not have an HIV test	ref	
Had an HIV test	0.79 (0.66-0.93)	
Living with HIV	1.67 (1.25-2.21)	
Interview conducted post-PCEPA	2.00 (1.72-2.32)	1.58 (1.35-1.85)

^a^Time-updated measures (serial measures at each study visit using last 6 months as reference point)

^b^Variable was included in multivariable analysis but was not retained in the best fitting model

### Objective 2: Independent associations between seeing mostly regulars and odds of experiencing workplace sexual violence and client condom refusal

In separate multivariable confounder models, seeing mostly pre-screened regulars was independently associated with reduced odds of workplace sexual violence (marginal association with *p*=0.067) and client condom refusal (Table 3) after adjusting for key confounders.

**Table 3:** Multivariable GEE independent associations between seeing mostly regular clients and workplace sexual violence and client condom refusal among sex workers in Metro Vancouver (n=925), AESHA 2010-2019

Exposure	Outcome: Experienced workplace sexual violence^a^		Outcome: Experienced client condom refusal^a^	
	Odds Ratio (95% CI)	Adjusted Odds Ratio (95% CI)	Odds Ratio (95% CI)	Adjusted Odds Ratio (95% CI)
**Saw mostly regular clients**^a^	0.60 (0.44-0.81)	0.73 (0.53-1.02)	0.66 (0.55-0.80)	0.70 (0.57-0.86)

^a^ Time-updated measures (serial measures at each study visit using last 6 months as reference point)

Both models adjusted for key confounders retained in the model fitting process, including age, non-injection drug use, homelessness, primary place serving clients (retained in workplace sexual violence model only), and whether the interview was conducted post-end demand law reform.

### Objective 3: Interaction between seeing mostly regulars and work environment on workplace sexual violence and client condom refusal

In multivariable GEE confounder models examining the additive interaction between seeing mostly pre-screened regular clients and work environment, participants who saw mostly regulars and primarily worked in outdoor/public or informal indoor venues faced significantly lower odds of experiencing workplace sexual violence (AOR 0.69, 95%CI 0.49-0.95) and client condom refusal (AOR 0.64, 95%CI 0.52 -0.80) (Table 4) relative to those who worked in the same venues and did not see mostly regulars. There was evidence of reduced odds of sexual violence and client condom refusal for sex workers who worked in formal indoor venues whether they saw mostly regulars or not, compared to those who did not see mostly regulars and worked in outdoor/public or informal indoor spaces, although the majority of these differences were not significant in multivariable analysis.

**Table 4:** Additive interaction between seeing mostly regular clients and work environment on workplace sexual violence and client condom refusal among sex workers in Metro Vancouver, Canada (*n*=925), AESHA 2010-2019

	Outcome: Experienced workplace sexual violence^a^	
**Exposure**	**Unadjusted Odds Ratio (95% CI)**	**Adjusted Odds Ratio (95% CI)**
Did not see mostly regular clients and primarily worked in outdoor/public or informal indoor spaces^a^	ref	ref
Saw mostly regular clients and primarily worked in outdoor/public or informal indoor spaces^a^	0.50 (0.37-0.69)	0.69 (0.49-0.95)
Did not see mostly regular clients and primarily worked in formal indoor venues^a^	0.26 (0.15-0.45)	0.55 (0.28-1.08)
Saw mostly regular clients and primarily worked in formal indoor venues^a^	0.19 (0.06-0.61)	0.41 (0.11-1.51)
**Exposure**	**Outcome: Experienced client condom refusal**^a^	
	**Unadjusted Odds Ratio (95% CI)**	**Adjusted Odds Ratio (95% CI)**
Did not see mostly regular clients and primarily worked in outdoor/public or informal indoor spaces^a^	ref	ref
Saw mostly regular clients and primarily worked in outdoor/public or informal indoor spaces^a^	0.57 (0.46-0.70)	0.64 (0.52-0.80)
Did not see mostly regular clients and primarily worked in formal indoor venues^a^	0.39 (0.28-0.55)	0.63 (0.41-0.95)
Saw mostly regular clients and primarily worked in formal indoor venues^a^	0.46 (0.27-0.79)	0.78 (0.43-1.44)

^a^ Time-updated measures (serial measures at each study visit using last 6 months as reference point)

Both models adjusted for key confounders retained in the model fitting process including age, non-injection drug use and homelessness^a^, and whether the interview was conducted post-end demand law reform

## Discussion

Over this 9-year community-based cohort study involving 925 women sex workers, over half of participants (57.3%) saw mostly regular clients at some point. Over the 9-year study period, 20.9% of participants reported experiencing sexual violence from clients: a lower proportion that in research from Baltimore which found that 22% of street-based sex workers experienced client-perpetrated violence in the past 3 months [42], and in Cote d’Ivoire where 30.6% of sex workers reported use of force or violence by a client [4]. 40.2% of participants faced client condom refusal: a similar proportion to studies from Australia in which 40.8% of sex workers faced demands for condomless sex from some clients [43], but lower than in Thailand where 71.5% of sex workers reported recent client condom refusal [44]. Those who saw mostly pre-screened regulars had reduced odds of facing workplace sexual violence and client condom refusal, highlighting how sex workers’ active screening and vetting of clients shapes clients’ behaviour and enhances sex workers’ safety and labour conditions within a criminalized context. The identified protective effect of seeing mostly regulars on sexual violence and condom refusal from our additive interaction sub-analysis was most significant among sex workers working in outdoor/informal indoor spaces. Under end-demand laws, participants had a 1.6-fold increased odds of seeing mostly pre-screened regulars (not fewer clients, but a greater proportion of pre-screened clients vs. new clients). Our findings suggest that seeing pre-screened, vetted regulars represents an important occupational health and safety strategy for sex workers which is rendered even more critical amid end-demand criminalization which has been documented to undermine sex workers’ ability to screen new clients [28, 31, 37, 45], and one which may be particularly salient in precarious work environments lacking the structural supports associated with formal indoor venues.

### Seeing mostly regulars and diverse modes of work

In our study, sex workers who were older and worked primarily in informal indoor spaces (e.g., apartments, vs. on the street/in public) had greater odds of seeing pre-screened regulars, while non-injection drug users and those who faced recent homelessness had lower odds of seeing regulars, highlighting important class differences in sex workers’ ability to see regular clients. While over half of participants saw mostly regulars at some point during the study, 42.7% reported never seeing mostly regulars, highlighting a diversity of modes of sex work. 30.8% of participants worked in formal indoor venues (e.g., massage parlours) at baseline and had significantly reduced odds of seeing mostly regulars (AOR 0.56, 95% CI 0.41-0.78), which is expected as many massage parlours are street-facing venues that receive higher numbers of one-time and walk-in clients. Formal indoor venues can provide a broad established clientele (i.e., clients who routinely visit the venue, negating the need for the worker to market their services/find their own clients), physical security, and managerial support with the labour of client screening [2, 36, 46–49] which can enable indoor workers to see more one-time or occasional clients relative to those who work in outdoor/public spaces who do not utilize the screening, security, and other supports of indoor venues (and thus may rely more on regular, pre-screened clients). Further, prior research has shown that some sex workers prefer short client interactions due to desire for anonymity and/or not wanting to engage in emotional labour, and thus organize their work accordingly (e.g., by offering time-limited sexual services in formal indoor venues) [50, 51], while others highlight romance, emotional connection and intimate conversation as services offered to clients, and thus structure their labour to enable longer-term client relationships [51]. In general, our results highlight diverse approaches to client type and work environments, suggesting that policy efforts to promote sex workers’ safety and sexual health including HIV/STI prevention should dismantle all restrictions which undermine sex workers’ screening abilities, and enable sex workers to structure their labour and select clients according to their own preferences.

### Impacts of seeing mostly regulars on client condom refusal and workplace sexual violence

Our study found that seeing mostly pre-screened regulars was associated with reduced odds of client condom refusal (coercion into any type of condomless sex) within a criminalized setting. Our findings differ in some ways from global literature: in Asia and sub-Saharan Africa, the client vs. intimate partner boundary has been documented to be more fluid, with implications for condom use. In the Dominican Republic [20], Madagascar [52], Uganda [53] Nepal [21] and Indonesia [22], repeat visits and greater relationship intimacy (i.e., viewing the client as more of a boyfriend) has been associated with lower condom use. However, our results align with research from western contexts where sex workers reported high rates of client condom use and minimal overlap between clients and non-paying partners. In Scotland, sex workers used condoms almost 100% of the time with clients and rarely with intimate partners [54], and a Canadian study found that 87.2% of sex workers and 90.4% of clients reported always using condoms in a sex work encounter relative to 40.1% and 8.6% always using condoms in romantic relationships, respectively [55]. Given the potential for HIV/STI exposure associated with condomless sex with many partners, there is some evidence that ‘hobbyists’ or clients who see sex workers often are more likely to use condoms: a US study found that regular clients were more likely to be risk averse and use condoms than were new (i.e., first time or second time) sex work clients [56].

Sex workers who saw mostly pre-screened regulars also had lower odds of facing workplace sexual violence (sexual assault or rape while working): a similar pattern as in research from Togo and Burkina Faso which found that among sex workers who experienced sexual violence, 34.5% reported that perpetrators were new clients relative to 18% reporting that perpetrators were repeat clients [57], and in Cote d’Ivoire where among those who reported sexual violence, perpetration by occasional or new clients accounted for the largest proportion at 37.2% (95% CI: 27.6 to 47.8), compared with regular clients who accounted for 18.1% (95% CI: 11.8 to 26.7) [4]. Further, in Thailand, odds of facing client condom refusal were higher among sex workers who experienced recent physical or sexual workplace violence (85.6% vs. 69.0% respectively; ARR 1.24, 95% CI 1.14-1.35) [44]. Despite prominent stereotypes positing sex buyers as unilaterally violent, research from North America and the UK suggests that male clients of sex workers generally reflect the broader population of men [58–60]; and reject sexual entitlement and ideologies that provocative women are deserving of violence [61, 62]. Our findings are consistent with evidence that sex worker-client interactions are diverse; can range from violent (in the case of aggressors posing as clients) to uneventful to mutually pleasurable [11, 61, 63] as in other relationships and sexual interactions; and critically, highlight sex workers’ agency in their work. Moving beyond traditional public health research and legislative approaches which conceptualize sex workers as passive victims facing risk of client-perpetrated violence and HIV/STIs, the association between seeing regulars and reduced odds of workplace sexual violence can also be understood as a result of client selection, screening, vetting and retention processes implemented by sex workers. Clients who are respectful of boundaries (i.e., services offered, condom use) are those able to be seen again and become regulars. Clients are typically re-vetted by sex workers at every visit, with workers actively filtering the characteristics of repeat clients, shaping decreased boundary violations (sexual violence, condom refusal) among pre-screened regulars relative to one-time clients as identified in our analyses. Applying this labour lens to sex workers’ occupational safety strategies enables vital acknowledgement of their expertise and active participation in shaping the organization of their labour and filtering their clients (similarly to other small business owners), which is rendered even more critical by end-demand criminalization which has been shown to undermine sex worker and client communications and screening [28, 30–32]. The use of this labour lens also affirms sex workers’ extensive advocacy efforts to resist against reductive frames of passive victimization, towards enhancing their rights [12, 36, 64, 65].

### Interactions between seeing mostly regulars and work environment on workplace sexual violence and client condom refusal

In additive interaction models, the protective effect of seeing mostly pre-screened regulars on workplace sexual violence and client condom refusal was most significant for participants working in outdoor/informal indoor spaces, suggesting that under criminalized conditions, sex workers may see pre-vetted regulars to enhance their occupational safety in more precarious workspaces. The reduced odds of sexual violence and condom refusal for participants who worked in formal indoor venues whether they saw mostly regulars or not is consistent with epidemiological and qualitative evidence that managed indoor settings often offer crucial workplace protections to sex workers [8, 15, 36]. Occupational safety supports in formal indoor venues can include security cameras; sexual health resources (i.e., education/condoms/lubricants); and the presence of venue managers, security, receptionists, and other sex workers to screen clients and monitor the space [2, 11, 36, 47, 63, 66, 67]. A Canadian study found working in managed indoor venues to be strongly associated with reduced HIV and STI prevalence and enhanced condom use among sex workers [68]. Despite the criminalization of third parties under end-demand laws in Canada and elsewhere, screening clients, intervening in and de-escalating conflict, and removing violent aggressors have been cited as critical roles of managers/security in indoor venues, which contribute vital oversight within the work environment and enhance sex workers’ physical and psychological safety [2, 3, 36, 69, 70]. As our previous research found that sex workers who saw mostly regulars had lower odds of accessing third party (i.e., venue manager, security) support [48], our findings suggest that ensuring that sex workers can engage third parties to assist in the labour of screening/vetting clients if they choose may enable sex workers to see more one-time clients. Our results and existing evidence suggest that working in formal indoor venues with security and screening supports may alleviate sex workers’ need to rely on regular, pre-vetted clients in order to work safely in criminalized conditions.

### Policy implications

Our study results highlight protective effects of seeing mostly pre-screened regulars within a setting where sex work is policed and clients are criminalized, underscoring a need for policies which enable sex workers to structure their labour according to their own preferences to ensure optimal occupational health. The ability to screen and see new clients is critical for many sex workers’ income security – as it is for other small business owners – and should not present health or safety risks. Our findings contribute to a body of evidence demonstrating that sexual violence and HIV/STI exposure in sex work interactions are not a result of clients being unilaterally predatory, but are shaped by broader structural determinants including criminalization, punitive policing, work environments [1, 8, 9, 29], and misogyny and racism which impact both clients’ and police’s interactions with sex workers [11, 62, 64, 71]. Across diverse settings, client criminalization has been shown to create greater pressure to accept clients’ terms, undermine sex workers’ negotiating power for condom use, and heighten their vulnerability to workplace sexual violence [6, 28, 31], highlighting how the structural violence of denying labour rights and protections to sex workers enhances workplace violence and rights violations against them.

Our study identified increased odds of seeing mostly pre-screened, regular clients (as opposed to one-time clients) post-end demand law reforms. This likely reflects the reality that under exacerbated criminalization which renders client screening more difficult, more sex workers are choosing to see clients who are pre-vetted and pre-screened. This finding is consistent with emerging evidence from Europe and Canada showing that end-demand criminalization has undermined sex workers’ ability to screen new clients [28, 31, 37, 45], as clients are more hesitant to provide personal information [28, 30–32] and third parties are criminalized for assisting sex workers with screening [36]. When Canada’s previous sex work laws were ruled unconstitutional in 2013, the Supreme Court of Canada determined that screening is one of the most significant ways that sex workers are able to protect themselves from potentially violent or undesirable clients [72]. However, under end-demand legislation implemented in 2014, 25% of sex workers reported reduced ability to screen and negotiate with clients [37]. In another study involving clients, clients explicitly cited only seeing sex workers they had seen before and giving the least screening information possible to un-vetted sex workers as strategies to avoid potential criminal charges [45]. This evidence and our findings suggest that *both* sex workers and clients may be prioritizing seeing pre-vetted individuals that they have seen before to mitigate the harms introduced by end-demand laws. Rather than leaving sex workers solely responsible for alleviating the structural vulnerability engendered by criminalization, legislative efforts to enhance their safety should dismantle all restrictive policies (i.e., client and third party criminalization) so that sex workers may organize their labour to create optimal conditions for occupational health. The full decriminalization of sex work would remove existing structural barriers to client screening processes, facilitating this vetting for sex workers across diverse work environments (whether they prefer to see mostly regulars or new clients [73, 74]) and advancing sex workers’ labour and human rights.

### Strengths and limitations

This study relies on observational data which cannot be used to infer causality; additionally, our analyses rely on self-reported data which may be subject to recall, social desirability, or misclassification biases. However, our frontline staff includes experiential (current/former sex workers) and community-based staff who build deep rapport with participants across interview and outreach activities, which is likely to mitigate social desirability bias. A strength is our study’s nuanced focus on sex workers’ agency and expertise, moving beyond more risk-oriented framings of past research pertaining to sex work clients. This includes the use of interaction models to further scrutinize variations in the relationship between seeing regulars and sexual violence and condom refusal outcomes across diverse work environments, which was informed by community feedback on preliminary results. A limitation is that our additive interaction term used a binary variable for work environment (i.e., formal indoor venue vs. outdoor or informal indoor space). While there are critical differences between outdoor and informal indoor workspaces, we grouped these due to the complexity of an additive interaction term with six categories. Though we hypothesized primary differences would be among formal indoor sex workers, we may have limited power to detect these differences due to the relatively small number of formal indoor workers who saw mostly regulars, and the non-significance of these results should be interpreted with caution. In addition, sex workers who were more mobile and those lost to follow-up could be at higher risk of sexual violence. However, we confirmed that number of study visits was not associated with sexual violence or condom refusal among this sample. Further research is recommended to explore potential differences in seeing regular clients among sex workers working in street-based and diverse informal indoor spaces, ranging from private apartments to clients’ places to supportive housing settings.

## Conclusions

Our study identified associations between seeing pre-screened regular clients and decreased odds of workplace sexual violence and client condom refusal, suggesting that within criminalized settings and precarious work environments which render client screening increasingly difficult, sex workers may see mostly regulars to enhance safety and sexual health, including condom use. Sex workers’ ability to see regular clients may represent an important occupational health strategy which should be supported at legislative levels by dismantling client criminalization which undermines sex workers’ screening strategies and agency. It is imperative that policy decisions on sex work regulation are based on empirical data about sex worker-client interactions rather than inaccurate misconceptions. While our findings suggest that sex workers are seeing pre-screened regulars to aid in mitigating the structural vulnerability engendered by criminalization, it’s unacceptable that current sex work laws undermine sex workers’ ability to work safely. Legislative efforts to promote sex workers’ occupational safety including HIV/STI prevention should target violence and coercion explicitly rather than adults engaging in consensual sex service exchange, as also recommended by international policy institutions including UNAIDS and Amnesty International [75, 76]. The full decriminalization of sex work, including removal of end-demand client and third party criminalization, is necessary to enable sex workers to communicate with clients, screen clients, and organize their work according to their needs, towards HIV/STI prevention and the full achievement of sex workers’ labour and human rights.

## Data Availability

Due to our ethical and legal requirements related to protecting participant privacy and current ethical institutional approvals, all relevant data are available upon request pending ethical approval. Please submit all requests to initiate the data access process to the corresponding author.

## Abbreviations

AESHA: An Evaluation of Sex Workers Health Access
GEE: Generalized estimating equations
HCV: Hepatitis C virus
HIV: Human immunodeficiency virus
IQR: Inter-quartile range
STI: Sexually transmitted infection
